# A unique approach to fostering student wellbeing while supporting community needs through an interprofessional, One Health, access to care veterinary clinic: WisCARES

**DOI:** 10.3389/fvets.2025.1602028

**Published:** 2025-06-03

**Authors:** Elizabeth E. Alvarez, Ruthanne Chun, Jennifer W. Brooks, Kelly Schultz, Simon Lygo-Baker

**Affiliations:** ^1^Department of Medical Sciences, School of Veterinary Medicine, University of Wisconsin-Madison, Madison, WI, United States; ^2^Center for Education, King’s College London, London, United Kingdom

**Keywords:** one health, access to care, veterinary education and research, social work, Veterianary, service learning and community partnerships, spectrum of care, contextualized care

## Abstract

Service-learning, defined as integrating student education into academically relevant service activities to address a community need, is a way for students to learn and practice multiple skills. WisCARES (Wisconsin Companion Animal Resources, Education and Social Services) is a service-learning clinic in which veterinarians, veterinary nurses, and social workers form an interprofessional team providing a unique educational opportunity within a One Health access-to-care clinic with care for both the veterinary patient and the client. In addition to hands-on experiences in spectrum of care medicine, veterinary students learn about poverty, homelessness, and social determinants of health, and how these impact clients’ decision making and ability to adhere to treatment recommendations. They also work with social workers to understand how moral stress and perfectionism can impact their physical and mental health and develop a self-care plan to address their own stressors. WisCARES’ goal is to help students develop into veterinarians who will be positive additions the profession by recognizing and challenging their own biases, and by consciously integrating access-to-care medicine into their future practice for the wellbeing of their veterinary team and the community they serve.

## Introduction

The growing need for access to quality veterinary care is well described. An estimated 29 million dogs and cats live in families participating in the Supplemental Nutrition Assistance Program (SNAP), with millions more in financially struggling middle-class households (1). Over the past 20 years, biannual Point in Time surveys conducted by the Dane County Homeless Services Consortium (HSC) document 600–800 unhoused people sleeping in shelters or encampments in Madison, Wisconsin. Less visible are the hundreds of people who live in their cars or motels or ‘couch surf’ in the homes of friends or acquaintances. Reports estimate that 5–23% of people experiencing homelessness own pets (2). The United Way’s Asset Limited Income Constrained Employed (ALICE) report states that 23% of Dane County families (roughly 57,519 households) are living paycheck to paycheck; this population is employed and earns above the federal poverty level but does not make enough money to afford a basic household budget (3). According to data on pet ownership published by the American Veterinary Medical Association (AVMA) (4), at least 50% of families within this socioeconomic bracket own at least one dog or cat and more than one out of four (28%) families with pets experienced a barrier, primarily financial, to veterinary care (1).

Veterinarians often experience the difficult challenge of wanting to improve animal welfare and relieve patient suffering with a client who is unable to afford the cost of veterinary care, leading to feelings of moral stress (5). “Moral stress” can be defined as “(t)he feeling of not being able to do what you believe to be ‘the right thing’ because of constraining personal, professional, organizational or client factors.” (6, 7) This can result in feelings of powerlessness, anger, and guilt because of the inability to align actions with values or beliefs (1, 8, 9). Another reported source of anger or frustration for veterinary professionals is when they perceive that they “(w)ork with team members who do not treat vulnerable or stigmatized clients with dignity and respect…” (10) When asked about work stressors, a survey of practitioners from 200 veterinary hospitals (11) indicated that discussing and disputing fees is considered the third most common source of stress among veterinarians, contributing to a moderate-to-substantial level of burnout (5). These financial or other limitations of animal owners can negatively impact veterinarians’ ability to practice medicine at a level consistent with their ethics and standards and ultimately affects veterinarian well-being. To combat these unavoidable experiences, learning skills developed in professions such as social work to help diffuse moral distress surrounding working with families with hardships and financial constraints must be considered in veterinary training (10).

Interprofessional practice occurs when professionals from different fields work and learn together to achieve a common goal. In healthcare, interprofessional practice aims to improve the quality, affordability and equity of healthcare services while also attending to clinician well-being (12). Interprofessional practice is an effective way to enhance One Health, an approach to healthcare that recognizes the interconnection between people, animals, plants and their shared environment (13). At WisCARES, veterinary professionals and social workers use their respective expertise to deliver an innovative educational program for students and meet the needs of human-animal families who struggle daily to obtain medical care, housing, food, and other basic needs (14–17). Preliminary data suggests that students participating in this service-learning model retain positive benefits for weeks after the experience (17). This interprofessional, One Health clinic is designed to teach veterinary students strategies for practicing access-to-care medicine, identifying and understanding moral stress, and keeping themselves emotionally healthy.

Interprofessional, One Health, service-learning clinic models have been part of medical school curricula for decades and are recognized as an effective form of teaching and learning. Service-learning is “a means of integrating theory to practice” that “combine [s] classroom learning with meeting real community needs.” (18) A common example of service-learning clinics is student-run human medicine interprofessional clinics located within communities of people living with low-incomes or homelessness. In addition to practical clinical skills development and improved knowledge of preventive medicine, students participating in interprofessional service-learning opportunities have shown improvement in their interpersonal skills, increased levels of compassion and empathy, teamwork, leadership, and professional confidence and growth (18–29). Similar to the student benefits recognized in other medical fields, early work in veterinary medicine indicates that service-learning community-based experiences improve the health and welfare of underserved companion animal patients (14, 27, 30). With the inclusion of an interprofessional social work focused curriculum, additional benefits beyond the student and the pet extend to the humans in the families bringing in those patients (17, 18, 31).

On an individual student basis, rubrics were developed from the approved CBVE standards (32). While the intention is for certain outcomes to be achieved and this is what the rubric is used to help the students be aware of, it is also the intention and experience that the learners gain more than this from daily verbal feedback and qualitative feedback at the end of the rotation. This is often dependent upon the previous experiences they have had, the values they arrive with and their expectations. Much of the feedback shows that the outcomes therefore range from consolidating previous experience of working with similar clients, to those who find themselves exposed and challenged to new and very different outcomes. Nearly every student has an obvious but often difficult to quantify developmental arch at WisCARES. Often this is evident in their ability to move through difficult cases with more ease and confidence.

Social workers are a key member of an interprofessional health care team in human in-patient and out-patient settings (33, 34). Over the past decade, social workers have been increasingly employed within veterinary schools. Most commonly, veterinary schools employ social workers, or other mental healthcare providers, to help students work through the significant demands of a pre-clinical veterinary curriculum (35). Other studies report the positive impact of adding social work topics into the curriculum during veterinary student clinical education (35, 36). The demands of the pre-clinical years are often vastly different than the stressors students face throughout their clinical year and after graduation. Factors associated with clinical year veterinary medical professionals’ distress include mismatched/unclear client expectations, unexpected outcomes, and emotional exhaustion (36, 37). Several authors suggest that veterinary students should be taught discrete skills in coping and self-care (35, 38–40). The 2018 edition of the Merck Animal Health Wellbeing Study, with responses from 3,600 veterinarians, concludes that “(g)iven the high levels of stress inherent in the veterinary profession…, we believe that it would be helpful for veterinarians to consult with a social worker or mental health professional to develop a stress management plan to help strengthen their ability to cope…” (36). Encouragingly, medical students engaged in self-care practices throughout their training experience a higher quality of life, greater resilience and lower risk for elevated levels of distress (41, 42).

Several veterinary outreach clinics leverage the human-animal bond and are “One Health Clinics,” where clients can access medical services for themselves, such as dental care, smoking cessation, and vaccinations (43–45). Additionally, effectively assisting animal owners coping with homelessness, addiction, domestic abuse, or mental and physical health concerns with the help of social work expertise can challenge students’ stereotyped perceptions of homelessness, pet-ownership and underserved populations (17, 18, 31). Students who participate in service-learning clinics with social workers benefit from both observing and learning social work skills and strategies for working with people, as well as the positive impact of service learning and volunteerism on one’s own mental health (44). The goal of this manuscript is to describe the WisCARES model by delving into the staff, student and client experience, as a way to help others find inspiration and avoid some of the pitfalls we experienced.

## Context

Established in 2013, WisCARES is a collaboration between the Schools of Veterinary Medicine, Pharmacy, and Social Work at the University of Wisconsin-Madison (UW). WisCARES aims to increase access to veterinary services for low-income and homeless pet owners, provide concurrent social services and human health care support to the same clientele, and engage participating students with practical interprofessional service-learning in a One Health setting. The program focuses on keeping pets with their owners, preventing surrender to animal shelters, empowering people to care for their animals, providing housing resources, and aiding animals and their people in gaining access to the social support services and healthcare they need. To receive services, pet owners must have incomes at or below 200% of the federal poverty level, be experiencing homelessness or require otherwise unaffordable veterinary medical services to avoid eviction or to obtain housing. One client, Nicole, shared her story in a JAVMA article in 2015: *“You know people are homeless, that homelessness exists. It blew my mind when it was me,”* said Nicole, 38, who asked not to use her last name. *“I’m the kind of person the safety net is supposed to catch: educated, middle-class. I’ve worked my whole life, I do not have any legal problems. I should not be sitting in a parking lot because I have no better place to go.”* Nicole adopted Emma, a German Shepherd-Siberian Husky mix 1 year before Nicole was diagnosed with a chronic illness which led to the end of her marriage, the loss of her job and the loss of her home. Fifteen years later, Emma is arthritic, and a kidney illness requires that she eat a special diet. Nicole says her disability payments aren’t enough to cover the veterinary bills*. “WisCARES has been a big blessing for me,”* she said, as the program provided Nicole with the prescription diet Emma needed. (JAVMA News, December 1, 2015, Vol 247, #11. Pp 1194–1199).

In addition to their income or housing status, the majority of clients are vulnerable for other reasons. For example, 40–50% of WisCARES clients report that they are people of color, 10–20% report being LGBTQA+ and 60–70% report having some form of disability (physical, cognitive, learning or emotional). These circumstances often reinforce one another, making it difficult for disadvantaged people to obtain the care they need for themselves and their animal companions. An interprofessional One Health approach provides care for both people and animals, protects the human-animal bond, treats and prevents zoonotic disease, and leads to better human health outcomes.

WisCARES is a full-service veterinary clinic, open 5 days a week, situated in a lower socioeconomic zip code area. The clinic provides preventative, routine, and urgent veterinary medical care, as well as dental care and surgical procedures. Undergraduate and veterinary student participation opportunities exist as a single-day volunteer experience to more consistent paid student hourly experiences for first year through third-year veterinary medical students; fourth-year students may enroll in two-week clinical rotations. Veterinary students work with clients to prioritize which diagnostic tests and treatment options to pursue, increasing their capacity to develop flexible diagnostic and treatment plans. Two full-time social workers provide resources and tools for students, clients, and staff as they work through complex care solutions. Throughout the rotation students participate in leading cases, debriefing, structured self-reflection, journaling and engagement with community members. Pharmacy, social work, and undergraduate students also participate at WisCARES, resulting in an interprofessional service-learning opportunity to provide compassionate care for the entire family unit, offering greater patient/client support and bridging potential gaps in care.

## Key programmatic elements

WisCARES has grown from a street medicine project led by a single veterinarian [funded by the UW School of Veterinary Medicine (UW-SVM)] to our current fully functioning interprofessional, community based One Health clinic. As the program developed, additional grant funding allowed for the hire of a full-time social worker. Initially, all services were provided at no cost to the client; the program was completely supported by donations of a variety of pharmaceuticals, pet food and pet supplies. Additional grants and monetary donations further supported the program with any additional work provided by volunteers and students. In this stage of program development, these funds, allowed the clinic to help a woman who was depressed and suicidal, but before she had herself hospitalized she wanted to know that her cat would be taken care of. The cat was cared for by WisCARES while the woman sought in-patient treatment for herself. (JAVMA News, December 1, 2015, Vol 247, #11. Pp 1194–1199). Early on, a pet boarding and foster program was initiated, to allow people to access their own in-patient medical care or safe housing without having to relinquish their companion animal.

In 2018, with financial support from the UW-SVM, WisCARES moved into 4,200 square feet building and became a full-service community veterinary clinic, with a tiered fee system. Because of the longer-term needs of most clients and the lack of adequate space for in-house boarding facilities, the boarding and fostering program pivoted to become exclusively an animal fostering program. WisCARES maintains, and builds new relationships with industry vendors that supply free or low-cost veterinary products. Major financial support for operations and curriculum development comes from numerous grants, providing the resources necessary to expand staffing. WisCARES is also supported by two 501c3 organizations, one dedicated solely to WisCARES and the other maintaining a WisCARES specific fund within a much larger 501c3 dedicated to supporting University programs.

WisCARES has a leadership team that is vital to the development and growth of the program. The team is comprised of six people who work collaboratively, with each having an equal say in decisions: (1) the current program director and administrator, is a veterinary specialist with over 10 years’ experience as a veterinary school hospital director, (2) the medical director and lead clinical instructor, is a veterinarian with multiple years of prior experience working in a high-volume access to care clinic, (3) a practice manager and lead CVT who joined the team after multiple years in general practice, (4) a social work and outreach director with over 20 years of experience in social work education and practice with people experiencing poverty and homelessness, (5) a curriculum director, who leads the grants and educational research and is also a veterinarian with 20 years’ experience teaching primary care and communication, and (6) a certified accountant and University grants manager who manages the program’s multiple sources of revenue by providing monthly oversight and annual budget forecasts. In addition to the leadership team, current clinic staffing includes two full-time veterinarians, a second full-time social worker, two additional CVTs, two full-time receptionists, roughly 8–10 student veterinary assistants, and several volunteer veterinarians. Since the beginning of the program, WisCARES has also had the input of an education specialist with over 25 years’ experience in teaching and research with adult education to support research and provide invaluable advice on curriculum development.

The veterinarians, social workers, and veterinary nurses at WisCARES are an interprofessional team that strives to implement the four core competencies of interprofessional healthcare teams, which include (1) working with a clear understanding of each other’s roles and responsibilities as well as (2) each profession’s values and ethics, while (3) striving for outstanding health team function (4) through clear and collegial communication (46). Clients often come with increased emotionality and varying abilities to comply with medical recommendations, requiring our social work and veterinary staff to routinely work together to mitigate client needs that are more complex than would be typically managed at a regular private veterinary practice. The social work concept of “person-in-environment” (47) provides a framework for thinking about how to address these situations. From this perspective, a client and their need are not viewed in isolation, but as existing within a milieu of social, economic, political, and educational systems (among others). Taking these systems into account is critical to developing treatment plans that people experiencing poverty and homelessness are able successfully carry out for their companion animals. WisCARES curriculum includes weekly rounds with social workers about the person-in-environment perspective, social determinants of health, self-reflection and awareness, personal/professional growth, and communication with clients. The curriculum is purposefully designed to support students’ abilities to respect and effectively communicate with clients facing poverty and includes specific concepts such as bringing awareness of cultural humility, also known as an understanding of one’s own identities and how they affect judgement, medical decision-making, and communication strategies.

## Discussion

In general practice, veterinarians are the surgeon, internist, ophthalmologist, cardiologist, nutritionist and much more for their patients. They often find themselves working to help their clients through difficult decisions in the lives of their pets as well. There is a tendency, particularly for those in health care, to want to ‘help’ families and sometimes this can extend beyond the boundaries of professional knowledge. This can be based on wanting to do the right thing but ultimately not having the requisite skill set. Having an interprofessional team means that the veterinarian is not the last resort for all conversations. For example, social workers’ training includes human behavior, mental health and counseling, social policies and progreams, and identifying social services to meet clients’ needs. Having a social worker as part of the veterinary clinic team creates the opportunity for discussions about client situations that are broader and include multiple persprectivers. Accessing a different professional resource demonstrates to veterinary students that they do not have to have all the answers. They learn how to leverage their resources, including the professionals around them, whether that be in their clinic or within their community.

Social workers and veterinarians are accustomed to working in settings where their respective professions determine priorities and provide services. From the communication rounds perspective at WisCARES, social workers bring a different and important perspective about working with people and finding ways to connect. It is important for students to hear the veterinary perspective side by side with the social work perspective. There is significant advantage to having an interprofessional component to this setting because it allows those undertaking different roles the opportunity to gain insight into a more holistic approach to the human animal bond and the complexity of each case that is seen. The opportunity to see how other professionals work, the language and symbols they use and recognize, allows those who do not share that identity an opportunity to gain greater appreciation of how the work they do can support the work of others. Successful care relies on a team approach and often the complex issues that clients face can be hidden from one team member or another. The opportunity to share experience, to learn and develop comes from the interprofessional environment.

The common frustrations with interprofessional practice also show up at WisCARES. Like any interprofessional practice, there is friction in working side by side on roles and responsibilities and values/ethics. The question of ‘who is in charge’ is a common point that needs to be clarified. For example, determining who oversees the scheduling of collaborative meetings and adherence to the guidelines set forth by members of each working group within the clinic can create some tensions without intentional communication. It’s often easier for the clinic team to understand and engage during meetings about the importance of how the veterinary clinic is functioning, etc. The challenge remains that the identities of each role are often difficult to elucidate, and yet they are important to each individual. It is a part of their profession and who they are, and a lack of clarity can become a barrier. At WisCARES the staff strive to understand each another’s professions and identify the mix of veterinary and social work services each family needs. Even with this intention, different perspectives bring assumptions about the needs and misunderstandings can arise unless these perspectives and assumptions are shared within the team. Another example of where misaligned team expectations can arise is whether the social workers function as veterinarians’ assistants, being brought into a client situation when the veterinarian thinks the client needs a social worker’s assistance, or whether social workers have their own practice domain and independently establish helping relationships with clients. Commitment to learning about each other’s training, scope of practice, and values and ethics helps WisCARES staff work through these kinds of questions. As the roles, and interprofessional environment become more embedded, these issues subside as shared symbols and ways of working together become more established.

Veterinarians and social workers working together can provide more comprehensive care for low-income human-animal families than either profession working alone. The two professions focus their interventions on different beings: veterinary medicine on the animal, and social work on the animal’s person. This difference, if approached with curiosity and openness, can be a source of expanded perspective and understanding for both professions. At times both may work with the family in a separate location and time, with the veterinary team providing pet vaccinations and the social work team providing information on housing resources. Other times, both professions work together more directly, such as participating in an emotionally aroused client’s appointment, each doing their part to help the client and patient and move the interaction forward.

Providing a platform for students to see and participate in community medicine is something that benefits both the community members and the students. As many as 60–70% of WisCARES clientele self-disclose a physical, mental, or emotional disability. Along with this, many WisCARES clients have higher levels of anxiety or more difficulty with cognition than a typical general practitioner may see in private practice. With every case having some degree of financial limitations, there are also times when everyone in the clinic feels judgmental and frustrated with the animal’s situation. Social workers participating in cases involving difficult conversations and limited resources can expertly model working through the frustration to understand the client and their situation so they can empathize and connect and do what they can to help and support the client.

Most WisCARES clients have needs beyond obtaining veterinary medical care for their pet, whether that is how to find a better paying job, learning the location of local food pantries, or listings of available rental housing. Many say that if they had not been approached by a social worker during their visit to the clinic, they would not have expressed those needs because it would not have occurred to them to talk to a veterinarian about them. For example, a client left this comment about their recent WisCARES experience: *“…(t)hey have been very kind & understanding without judging on one’s situation. They even have a social worker on hand to give out other resources info for those in need.”* Having social workers who can provide support and resource referral to meet human needs ensures that the entire human-animal family is cared for at WisCARES. Sometimes, the in-depth histories given by owners reveal deeper troubles than veterinary students can (or should be able to) handle. Students can share these conversations with the social work team, which can either work directly with the client or strategize with the student to develop a plan for the student to carry out themselves. This allows the student to see what it is like to participate in an exchange of expertise with another professional and also experience the value of shared, interprofessional, working.

Several times during each rotation, social workers meet with veterinary students to discuss client-directed topics like poverty, homelessness, social determinants of health, and communication, as well as wellbeing topics like moral stress, self-care, stress management, and self-compassion. Veterinary students are frequently surprised to learn that social work and other professions experience moral stress and that tools are available to effectively address it. Similarly, when accurate information about suicide risk is presented (48), existing beliefs about veterinarians’ unhappiness and vulnerability are challenged and, at times, re-evaluated. Personality traits common to veterinary students, such as perfectionism, give students the drive to gain admission to veterinary school while at the same time increasing the likelihood that they will experience struggles with mental and physical health (49–52). As students discuss these unrealistic standards with one another and WisCARES’ social workers and veterinarians, their emotional weight becomes clear, and many express interest in finding a different metric for evaluating the quality of their work. While having these discussions over the rotation’s 2 weeks, students also see patients and interact with clients in an environment that is designed to be both challenging and supportive. Through daily case debriefs, they realize that the world does not come to an end when they are not perfect and make a mistake. At times, there is resistance to these ideas, but students typically express relief knowing that that they can influence their own thinking and feelings through self-awareness and a deliberate approach to wellbeing.

While participating in the WisCARES interprofessional experience, students also speak of the value of seeing that they are part of a team and how important this is for effective practice. For example, a quote from a student survey response included: *“(t)his rotation helped me figure out how to work with multiple different disciplines. It helped me see that each person is important in providing the best quality care for everyone”* (17). This is an important learning opportunity, because it challenges the individuality of assessment and learning that is the focus for so much of formal veterinary education. Students at WisCARES see that while they often lead a case in practice and will be under pressure, they can rely on others for support and advice. The interprofessional topic rounds help students to discuss these issues and hopefully gain greater understanding of broader perspectives and dimensions of care. Nearly every student will encounter clients experiencing poverty on a daily basis. Educators at WisCARES are training students to structure their thoughts, develop coping strategies and utilize techniques for sound medical practice that will positively affect them for their whole career.

Providing veterinary medical care in a low-resource setting differs vastly from the traditional teaching hospital with its myriad available referral services. The day-to-day clinical functions at WisCARES more closely approximate general practice than a tertiary specialty service, and students report that they come away from the rotation with dramatically increased skills around general clinical workflow, communication and leadership (17). WisCARES research has also found that participating students from veterinary, pharmacy, and other programs report an increased awareness of the need for all patients to have access to basic and consistent veterinary care and a greater understanding of the spectrum of care concept (17). Students further recognize that clients’ economic constraints, abilities, and cultural beliefs affect treatment diagnostics and plans and students who worked with the social worker were more likely to feel comfortable working with people who were low income, homeless, or of a different race or ethnicity (17). These students were also likely to retain these positive feelings 1 month after their experience at WisCARES (17).

Multiple surveys and interviews, student evaluations and anecdotal conversations over the past decade show that students value the WisCARES experience and believe it will be helpful in their career. Student feedback about the program has been a key element to then enhance the experience. Much of the student feedback has supported the approach taken, for example, allowing students to take more responsibility: *“We were trusted to work independently and develop our own ideas, have our own conversations, and lead our own teams.”* However, students have also noted that *“being thrown into managing cases in a very different environment to the hospital is stressful.”* This transition has to be a supported approach and we have constantly revisited the curriculum to ensure that at the start of the rotation we make the students feel supported and able to work more independently. The curriculum continues to evolve based on what the students feel worked for them, such as the impact of learning and working with clients, and the importance of medical and social work debrief. A constant in the feedback is the value attached to the open and supportive approach in which the learners feel empowered to act at WisCARES. Students see a lot of medical cases. In any two-week rotation, they will see cases that vary widely in medical severity, ease of conversation with owners and moral distress regarding client or patient situations. If students were presented with these cases as hypothetical case examples or had a stronger leading hand from staff, the impact of these factors would likely be less. However, students at WisCARES face those situations head on with real members of their community and therefore feel the full impact. Students have this experience in an educational setting in which there are multiple safety nets. Even so, sometimes students are stressed by a case or overburdened. Another key skill they learn at WisCARES is that social workers and mental health professionals can help them identify ways to express themselves and ask for assistance during difficult times.

There remains much to explore regarding stress, coping, self-care, resilience, and mental health of veterinary students. As students participating through WisCARES have reported that *“(t)his rotation helped me find the spark and joy that is in me for veterinary medicine”* (17), we hope to not only continue this program for future decades, but also expand its influence. Sharing our findings and adding to the knowledge base by writing peer reviewed papers results in our program having a larger impact in the world. Further investigation is underway to assess if the long-term affect of these experiences, lessons and skills support veterinary well-being in future practice. One of our goals is to continue to work collaboratively with other programs to provide better access to care to all pet owners, while recognizing that the WisCARES model may not fit every location and that others can develop programs that work for their own learners and community.

## Data Availability

The raw data supporting the conclusions of this article will be made available by the authors, without undue reservation.
